# Case Report: Clinical case of a giant plexiform neurofibroma of the liver in a patient with deletion of exon 1 of the NF1 gene

**DOI:** 10.3389/fonc.2026.1771419

**Published:** 2026-06-04

**Authors:** Zhannat Idrissova, Madina Orazgaliyeva, Kristina Kovaleva, Zhanel Seilkhanova, Farida Rakhimbekova, Madina Zhaksybek, Dariga Myrzamuratova

**Affiliations:** 1 Competence Center for Neurological Orphan Diseases, University Clinic Aksai, Asfendiyarov Kazakh National Medial University, Almaty, Kazakhstan; 2 Center for Molecular Genetic research in Kazakh Institute of Oncology and Radiology, Almaty, Kazakhstan; 3 Genomic Laboratory, Asfendiyarov Kazakh National Medial University, Almaty, Kazakhstan; 4 Department of Neurology, Asfendiyarov Kazakh National Medical University, Almaty, Kazakhstan

**Keywords:** liver, MEK inhibitor therapy, MRI, *NF1* = neurofibromatosis type 1, plexiform neurofibroma

## Abstract

Neurofibromatosis type 1 (NF1) is an autosomal dominant disorder caused by mutations in the NF1 gene, including exon deletions, which are associated with various phenotypes and plexiform neurofibromas. Plexiform neurofibromas are clinically observed in up to 30% (and up to 50% on MRI) of NF1 patients and may involve both external and internal tissues, with a risk of malignant transformation to malignant peripheral nerve sheath tumors (MPNST). This report presents a rare case of a large plexiform neurofibroma in an adolescent male with NF1, involving the hepatic hilum and surrounding hepatic vessels. The patient, born in 2002, presented with multiple café-au-lait macules, cutaneous neurofibromas, axillary freckling, and headaches. Imaging studies, including 3-Tesla MRI and multislice CT, revealed a multinodular lesion extending along the hepatic vessels, around the gallbladder, the celiac trunk, and adjacent pancreatic and duodenal structures. Genetic testing confirmed a large deletion of an exon in the *NF1* gene. Due to the lesion’s inoperable location and risk of vascular and biliary compression, targeted therapy with the MEK inhibitor selumetinib was indicated. The patient is currently awaiting provision of the medication. This case underscores the importance of careful monitoring and early initiation of targeted therapy in NF1 patients with extensive plexiform neurofibromas, particularly those caused by large *NF1* gene deletions, which result in complete loss of neurofibromin and extensive infiltrative benign tumor growth.

## Introduction

Neurofibromatosis type 1 is an autosomal dominant disorder with complete penetrance, associated with various types of mutations in the *NF1* gene, including deletions with complete loss of protein function as well as single-nucleotide missense substitutions (1). However, up to 5–10% of cases are associated with large deletions involving entire exons of this gene, which lead to the most severe manifestations and are likely associated with plexiform neurofibromas. Plexiform neurofibromas (PNF) occur clinically (visible) in up to 30% and in 50% in MRI images. PNF may subsequently undergo malignant transformation, especially large lesions with pronounced infiltrative growth. Commonly, plexiform neurofibromas have both an external component (on the face, trunk, or extremities) and a deep component involving internal tissues or organs, usually with a visible external part. Alternatively, extensive plexiform neurofibromas of internal parenchymal organs are observed (1–6). Also, increased risk for additional tumors and mortality associated with symptomatic PNFs (7).

The aim of this study is to present a rare clinical case of a large plexiform neurofibroma in neurofibromatosis type 1, with spread around the hepatic vessels, in an adolescent with a large deletion of an exon of the *NF1* gene.

## Materials and methods

The patient was referred by a district oncologist to participate in a grant-funded study of the Ministry of Science and Higher Education of the Republic of Kazakhstan (grant No. AP19676226, Study of genetic markers and environmental factors in phakomatoses and neurogenic tumors). The patient was enrolled and followed up within the framework of this grant, informed consent of patient was obtained.

Diagnostic evaluation included genetic testing of the *NF1* gene using the MLPA method, imaging studies such as 3-Tesla magnetic resonance imaging (MRI) of the abdominal cavity, 1,5-Tesla brain MRI, multislice computer tomography (MSCT), liver fibroelastography, general clinical data, and neurological examination.

### Case description

**Figure 1:**
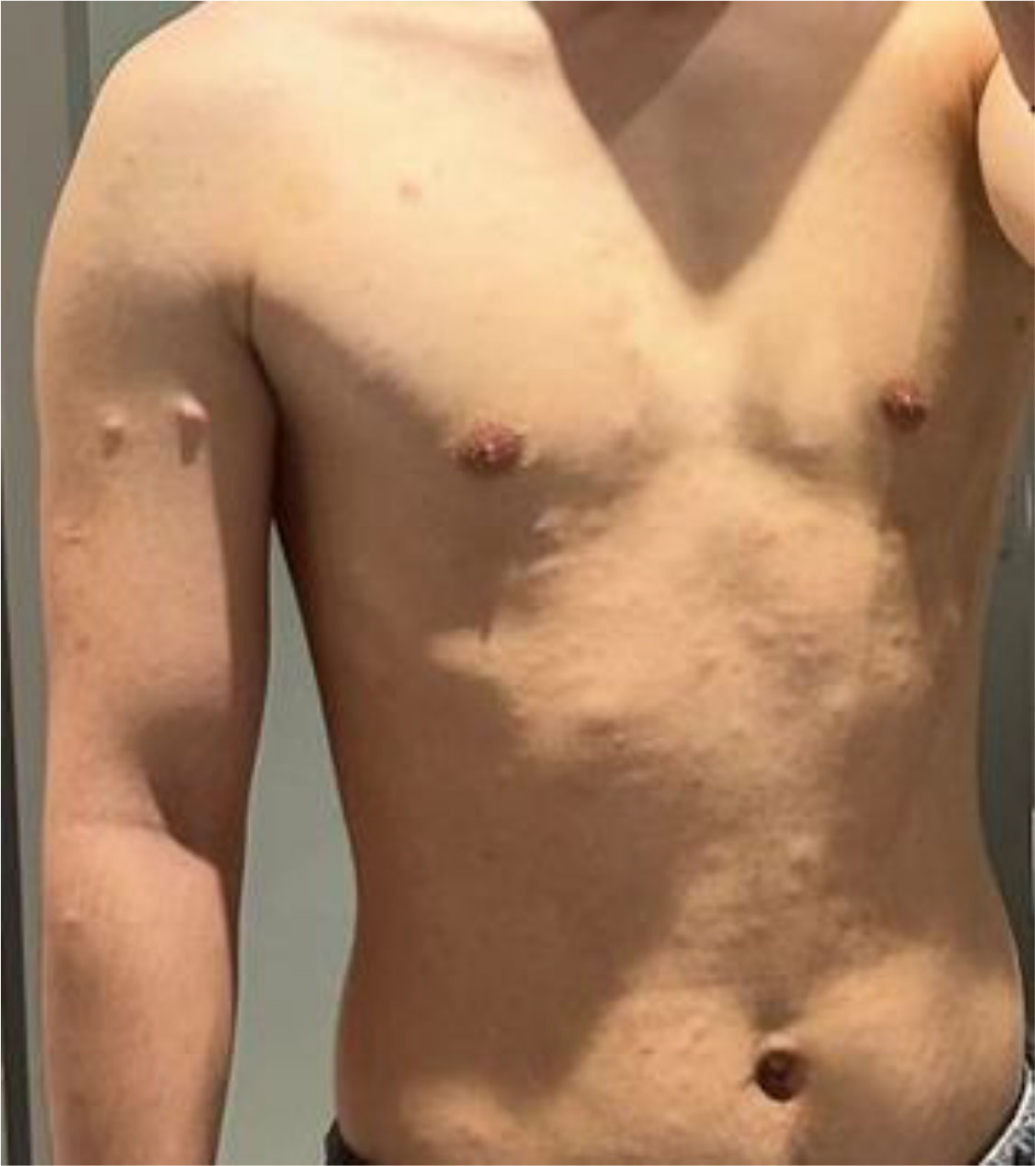
Café-au-lait macules and cutaneous (simple) neurofibromas on the patient’s body.

A 23 year old man presented with complaints of multiple café-au-lait macules (more than 50), the presence of cutaneous neurofibromas on the abdomen, axillary freckling (i.e., meeting the revised NF1 diagnostic criteria (1)), as well as headaches (**Figure 1**). Further, the diagnosis was proved by MLPA deletions encompassing total 1-st *NF1* gene exon deletion. Prior to referral to our clinic, abdominal ultrasound revealed nodular lesions in the liver, prompting liver ultrasound-based fibroelastography performed. In the liver parenchyma, lesions were detected in 7 of the 8 liver segments, with liver stiffness of 4.73 kPa, corresponding to METAVIR fibrosis stage F0. Timetable of the procedures performed for diagnosing of the case presented in **Table 1**.

**Table 1:** Clinical timeline for diagnostic workout.

Date	Event	Findings	Clinical decision
At presentation	Neurologist consultation (initial observation)	Presence of more than 50 café-au-lait macules >15 mm in diameter Simple neurofibromas on the skin of abdomen (>15) Headache Heaviness in the right upper quadrant, bitter burping.	Neurofibromatosis type 1, needs genetic testing
1 week after presentation	Biochemical parameters (venous blood)	AST 54 U/L (reference value <37–40 U/L), ALT 45 U/L Alpha fetoprotein 6 ng/ml (reference value < 10 ng/ml)	Considered liver pathology
2 weeks after presentation	Abdominal ultrasound	Nodular lesions in the liver	Confirmed liver pathology
3 weeks after presentation	Liver ultrasound-based fibroelastography	Lesions were detected in 7 of the 8 liver segments, with liver stiffness of 4.73 kPa, corresponding to METAVIR fibrosis stage F0.	Further confirmation of liver damage
1 month after presentation	Oncologist consultation (further follow-up)	Oncologist evaluated previous laboratory and instrumental findings	Neurofibromatosis type 1, plexiform neurofibroma in liver, need genetic testing
1 week after oncologist consultation	Genetic MLPA test for Neurofibromatosis type 1	Single exon deletion (1^st^ exon) in the *NF1* gene	Diagnosis neurofibromatosis type 1 confirmed
1 month after oncologist consultation	MSCT of the abdominal cavity	The region of the hepatic hilum showed a space-occupying lesion with irregular contours and a density of 26 Hounsfield units was identified, with overall dimensions of approximately 111 × 48 × 76 mm	Confirmation of neurofibroma ingrowth
1 month and1 week after oncologist consultation	3-Tesla MRI of the abdominal cavity	Hepatic multiple plexiform neurofibromas (clearly visualized on STIR sequences)	Further confirmation of diagnosis
2 months after oncologist consultation	Diagnostic conclusion	Plexiform neurofibroma of the liver, spreading along the biliary ducts arising from the celiac trunk	Admit target therapy – selumetinib
6 months after presentation	FDG PET of the body with 18F-FDG	Nodular lesions in the liver with SUV max 2.0	Suggesting the absence of malignancy
At the time of this manuscript writing	Therapy admission	Awaiting the start of therapy	

Biochemical parameters were as follows: AST 54 U/L (reference value <37–40 U/L), ALT 45 U/L (reference value <41–50 U/L), alkaline phosphatase 195 U/L (44 to 147 U/L), total bilirubin 20 µmol/L (reference value 3,4-20,5 µmol/L), direct bilirubin 1,5 µmol/L (reference value 1,5-5,1 µmol/L). These slightly elevated AST with complains to heaviness in the right upper quadrant and bitter burping turned us to perform abdominal ultrasound, results suggested liver ultrasound-based fibroelastography. In this stage we send patient to oncologist, who made the decision to conduct multislice computer tomography of the abdominal cavity, and then according to results to perform abdominal MRI, with focus to the liver. In parallel, a blood sample was collected from the patient for genetic testing (MLPA of NF1 gene), single exon deletion (1^st^ exon) in the *NF1* gene was detected.

Given the positive revised NF1 diagnostic criteria for neurofibromatosis type 1 and conclusion of oncologist, multislice computer tomography (MSCT) of the abdominal cavity was performed using a primary collimation of 128 × 1.0 mm, a reconstructed slice thickness of 1.5 mm, followed by three-dimensional image analysis with MPR, MIP, and VRT reconstructions. MSCT of the region of the hepatic hilum showed a space-occupying lesion with irregular contours and a density of 26 Hounsfield units was identified, with overall dimensions of approximately 111 × 48 × 76 mm. The lesion extended along the hepatic vessels of the right lobe of the liver and around the gallbladder. The remainder of the liver had smooth contours, was not enlarged, and showed homogeneous parenchymal density up to 60 Hounsfield units. The intrahepatic and extrahepatic bile ducts were not dilated. The common bile duct was not dilated. The gallbladder was ovoid in shape and without pathological changes.

**Figure 2:**
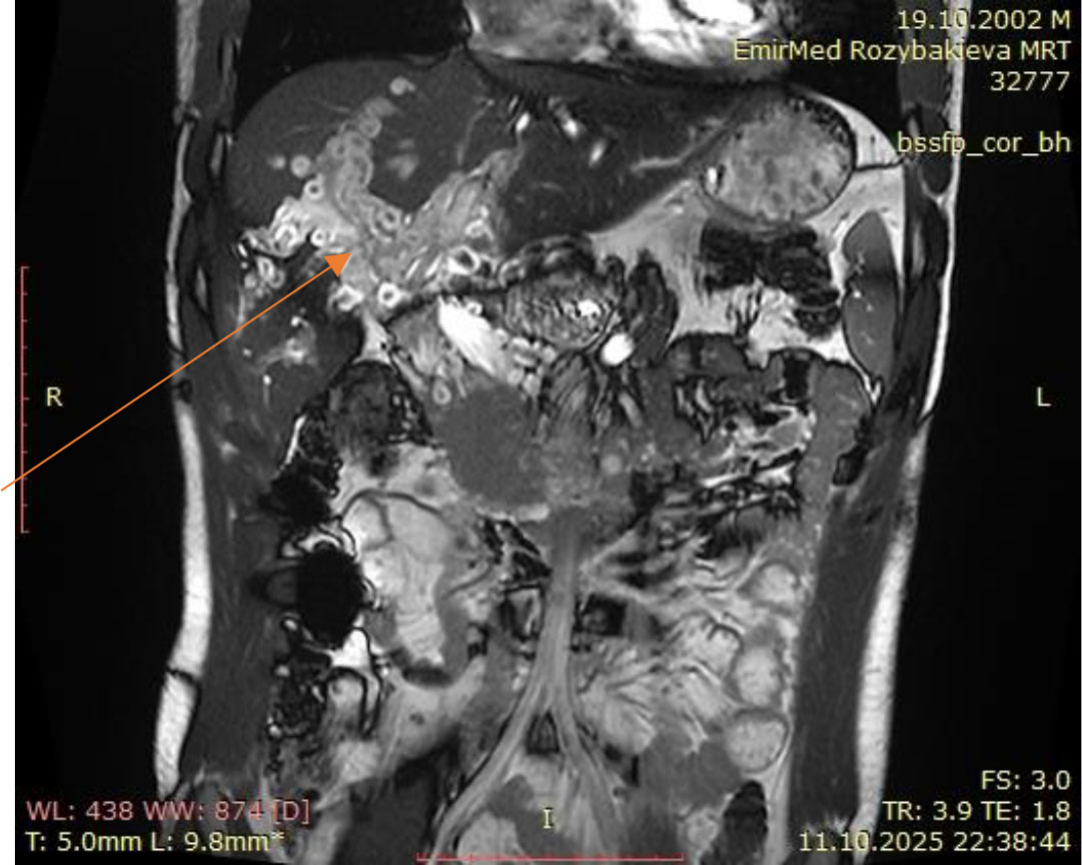
Targeted NF1 lesions in the liver originating from the celiac trunk in the patient on 3 Tesla MRI (5 mm slice thickness) in Fat Saturation (FS) regime.

**Figure 3:**
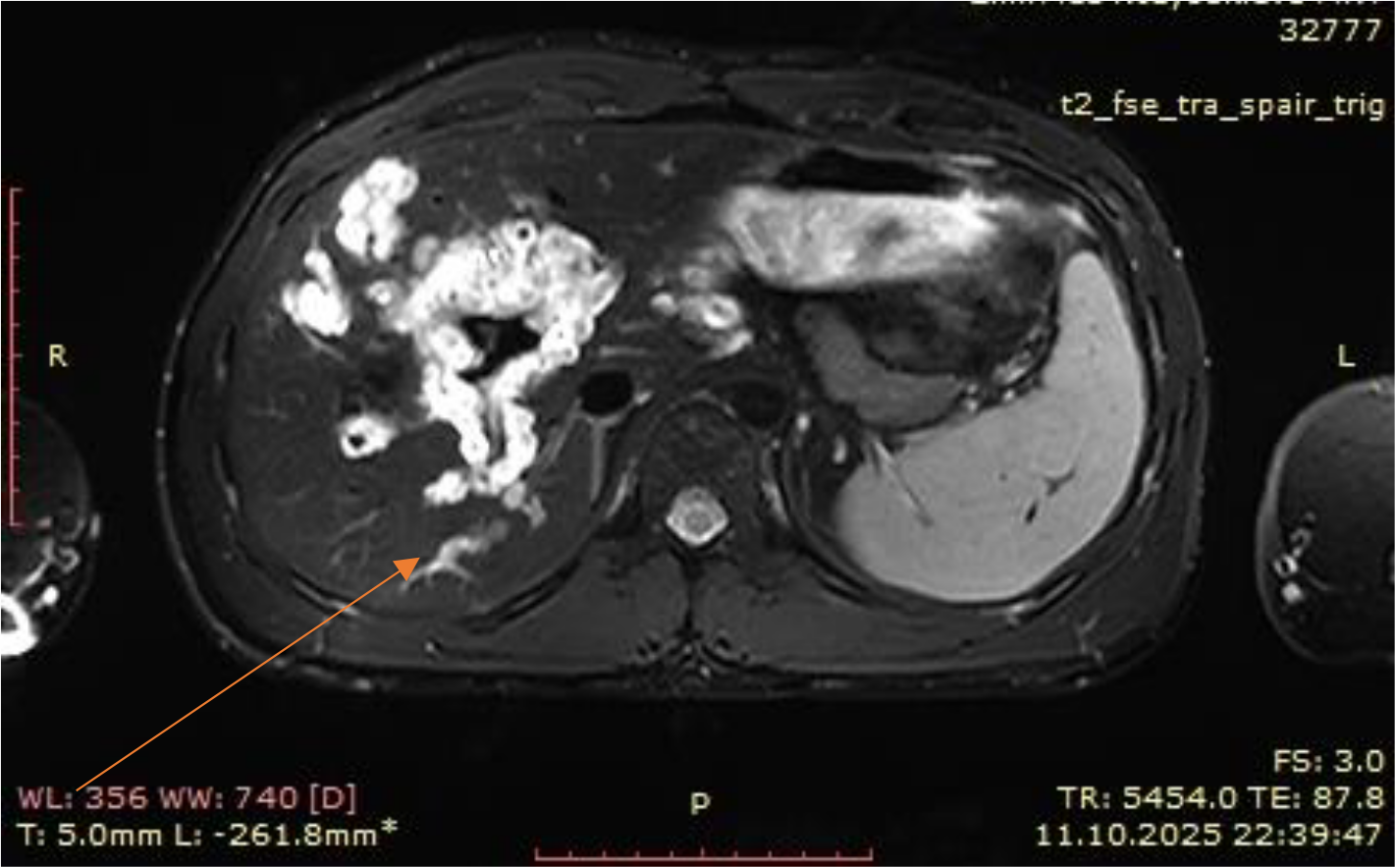
Extensive plexiform neurofibroma of the right lobe of the liver in the studied patient on 3 Tesla MRI (5 mm slice thickness) in Fat Saturation (FS) regime.

Subsequently, a decision was made to perform 3-Tesla MRI of the abdominal cavity and the brain. MRI revealed signs of a multinodular mass lesion in the hepatic hilum with extension along the vessels of the right hepatic lobe, around the gallbladder, along the celiac trunk, involving the head and body of the pancreas, and along the vessels of the duodenum—findings consistent with multiple plexiform neurofibromas (clearly visualized on STIR sequences) (**Figures 2**, **3**). The lesion originated from the nerve plexus of the celiac trunk. Diffusion-weighted MRI (DW-MRI) with ADC mapping was high (2,2×10^−3^ mm^2^ s^−1^) and the lesions demonstrates NF1 specific multiple target signs (Bull’s eye sign) on T2-weighted imaging, findings that support the diagnosis of plexiform neurofibromas (PN) rather than malignant peripheral nerve sheath tumors (MPNST).

Brain MRI visualized typical for neurofibromatosis type 1 FASI (Focal Areas of Signal Intensity) in thalamus and cerebellum.

At the time MLPA results identified 1-st exon *NF1* gene deletion.

Fluorodeoxyglucose Positron Emission Tomography (FDG PET) of the body with 18F-fluorodeoxyglucose (18F-FDG) was done recently (6 months after first presentation) and revealed lesions in the liver with SUVmax 2.0, which suggesting the absence of malignancy. Functional MRI using DWI comparable to FDG-PET for the characterization of PNFs in NF1 patients (8–10).

### CARE assessment

Diagnostic methods are listed in the **Table 1**. Diagnostic challenges included unusual localization (liver), lack of biopsy due to vascular risk, and the need to differentiate from malignant tumors. Differential diagnosis included malignant peripheral never sheath tumor (MPNST), hepatic lymphoma and other soft tissue tumors. Diagnostic reasoning included consideration of apparent diffusion coefficient – suggested benign lesions; implementation of Legius criteria; genetic testing.

## Discussion

As a result, the following clinical diagnosis was established: neurofibromatosis type 1, genetically confirmed; plexiform neurofibroma (space-occupying lesion) of the hepatic hilum and along the hepatic vessels on the right. This case is similar to observation of Yokogawa Y et al.2023 (11) who described giant diffuse plexiform neurofibroma invading the liver, also begin with minimal symptoms (practically asymptomatic).

The diagnostic algorithm is similar, however in this case there was not performed biopsy (which is limitations of the approach used), but we performed 3-Tesla MRI where typical target focuses of extensive plexiform neurofibroma of liver was demonstrated and genetic prove of *NF1* gene huge deletion also proved diagnosis (which are strengths of this clinical case).

Given the presence of an inoperable plexiform neurofibroma of the hepatic hilum, the risk of compression of the hepatic vessels and bile ducts, and the genetically confirmed diagnosis, initiation of targeted pathogenetic therapy with the MEK inhibitor selumetinib is clinically indicated (12–15). The patient is currently awaiting provision of the medication by the local outpatient clinic in 2026. Surgical intervention is not appropriate in this case due to multilateral growth of plexiform neurofibroma penetrating the liver. Liver transplantation is not indicated, including taking into account the patient’s preserved liver function.

The patient is being closely monitored by an oncologist, and at the time of this manuscript, remains stable, awaiting approval for initiation of MEK inhibitor therapy.

## Conclusion

Thus, the presence of a large deletion of an entire exon of the *NF1* gene, located at the beginning of this gene on chromosome 17, likely resulted in complete loss of neurofibromin synthesis and the development of a large plexiform neurofibroma with infiltrative growth in the liver, while remaining benign. This condition requires careful follow-up and prompt initiation of targeted therapy with MEK inhibitor selumetinib (12–15), which FDA (USA) approval granted in 2025 (and this is the only medicine approved for plexiform neurofibromas in NF1 in Republic of Kazakhstan).

## Data Availability

The original contributions presented in the study are included in the article/supplementary material, further inquiries can be directed to the corresponding author/s.
